# Supplementary material for: Causal interplay between lactose intolerance and gut microbiota: a combined bidirectional Mendelian randomization and in vivo validation study
Source: Front Nutr. 2026 Jun 1;13:1803337. doi: 10.3389/fnut.2026.1803337 (PMC13265576; doi:10.3389/fnut.2026.1803337)
Supplement: Supplementary file 1 [file Data_Sheet_1.zip › supplementary materials/Forward/leave one out cross validation/ebi-a-GCST90027485.finngen_R12_E4_LACTONAS.pdf]

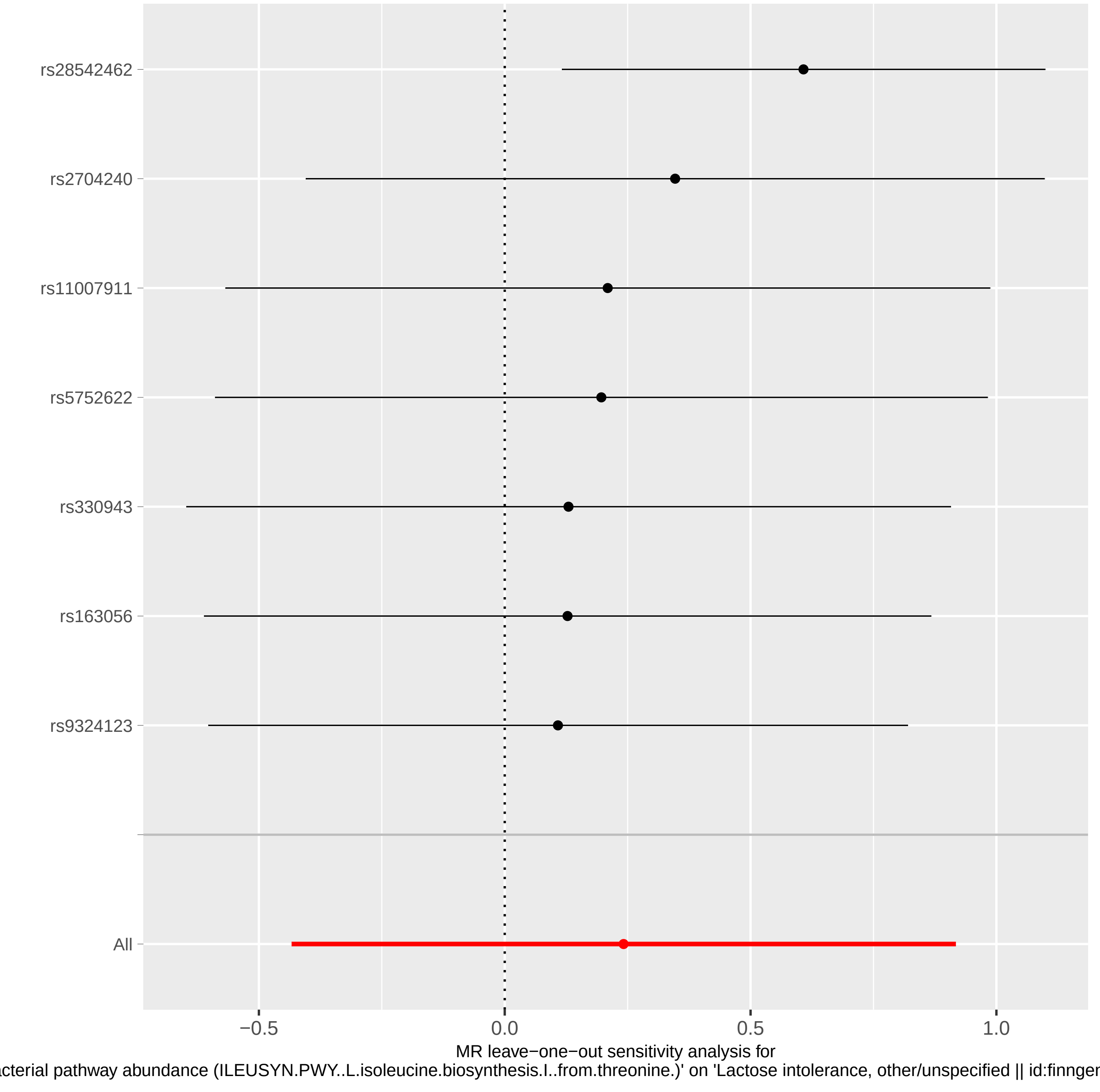

rs28542462
rs2704240
rs11007911
rs5752622
rs330943
rs163056
rs9324123
All
−0.5
0.0
0.5
1.0
MR leave−one−out sensitivity analysis for
acterial pathway abundance (ILEUSYN.PWY..L.isoleucine.biosynthesis.I..from.threonine.)' on 'Lactose intolerance, other/unspecified || id:finnger
